# Value of ^18^F-FDG PET/CT Combined with Lung HRCT in Diagnosis of Solitary Pulmonary Intravascular Metastasis

**DOI:** 10.1155/2022/8968855

**Published:** 2022-02-21

**Authors:** Yu Ji, Yaru Wang, Jing Song Zheng, Chun Chun Shao, Yong Cui

**Affiliations:** ^1^Department of Radiology, The Second Hospital Cheeloo College of Medicine, Shandong University, Jinan 250033, Shandong, China; ^2^Department of PET/CT Center, Shandong Cancer Hospital and Institute, Shandong First Medical University and Shandong Academy of Medical Sciences, Jinan 250117, Shandong, China; ^3^Department of Hospital-Acquired Infection Control, The Second Hospital, Cheeloo College of Medicine, Shandong University, 247 Beiyuan Rd, Jinan 250033, Shandong, China

## Abstract

**Background:**

Solitary pulmonary intravascular metastasis is a rare complication of malignant tumors, and accurate diagnosis can improve clinical decision-making, but diagnosing it effectively using conventional techniques is difficult.

**Purpose:**

To explore the value of ^18^F-FDG PET/CT combined with lung high-resolution computed tomography (HRCT) in the diagnosis of solitary pulmonary intravascular metastasis.

**Methods:**

^18^F-FDG PET/CT, lung HRCT, and follow-up data of 18,143 cancer patients were retrospectively analyzed to select patients with pulmonary vessel involvement besides the primary tumor only. The histopathological or imaging follow-up results were used as the diagnostic criteria for pulmonary intravascular metastasis.

**Results:**

A total of 13 patients with 17 pulmonary intravascular metastases were found, of which 9 patients had a single lesion and 4 had double. The SUVmax was 1.1–5.4 (average, 2.4 ± 1.4), and the length of hypermetabolic metastasis was 5.1–24.1 mm (average, 10.7 ± 6.5 mm). All the intravascular metastases were located in the terminal pulmonary vessels. Strip or branched pulmonary vessels enlargement with increased metabolism was the main imaging manifestation (15/17, 88.2%), while the other 2 cases only showed strip metabolic enhancement without abnormalities in pulmonary vessels (2/17, 11.8%). Four pulmonary intravascular metastases were confirmed by pathology, and the other 13 cases were diagnosed by imaging follow-up.

**Conclusion:**

^18^F-FDG PET/CT combined with lung HRCT is an effective technique for the diagnosis of solitary pulmonary intravascular metastasis. High-strip or branched FDG uptake in the distal pulmonary vessel accompanied by corresponding morphological changes in patients with malignant tumors can be used as a specific diagnostic indicator.

## 1. Introduction

Solitary pulmonary intravascular metastasis refers to the metastasis and proliferation of tumor cells along vascular endothelium without involvement of other parenchymal organs in cancer patients. Pulmonary intravascular metastasis is a rare form of lung metastasis. In most cases, tumor cells transport with the blood to slender pulmonary capillaries and adhere to capillary endothelial, and the tumor cell-endothelial cell interaction destroys the endothelial regulatory barrier and makes tumor cells exudate into the surrounding lung tissue. It can also be directly transferred to the surrounding lung tissue through transcellular migration to form intrapulmonary metastases [1, 2]. However, a small number of endothelium-attached tumor cells proliferate intravascularly, spread along the blood vessels and give rise to metastatic foci without extravasation. Al-Mehdi et al. also confirmed this special model for pulmonary metastasis [3].

Due to the slight clinical and traditional imaging manifestations, diagnosis of solitary pulmonary intravascular metastasis can be missed easily. The accurate diagnosis of solitary pulmonary intravascular metastasis is of great value in staging, early treatment, and prognosis evaluation of cancer patients and helps clinicians make decisions for tumor patients [4, 5]. At present, no study had systematically explored the imaging manifestation of solitary pulmonary intravascular metastasis, which is probably due to the rarity and few definite diagnostic methods of solitary pulmonary intravascular metastasis. ^18^F-fluorodeoxyglucose positron emission tomography/computed tomography (^18^F-FDG PET/CT) imaging can identify the malignant lesions at the functional level, which opens a new horizon for differentiating benign from malignant intravascular lesion. Studies have confirmed that ^18^F-FDG PET/CT can accurately diagnose malignant lesions in vessels, and the accuracy is close to 100% [6–9]. High-resolution computed tomography (HRCT) can accurately display the morphology of the pulmonary vessel, which is helpful for early detection of lesions.

This study retrospectively analyzed the ^18^F-FDG PET/CT and lung HRCT findings of 13 patients with solitary pulmonary intravascular metastasis and preliminarily summarized the metabolic and morphological characteristics of solitary pulmonary intravascular metastasis in order to improve the understanding for clinicians and radiologists.

## 2. Material and Methods

### 2.1. Patients

A retrospective analysis of the data of 18143 cancer patients was made, and they underwent both ^18^F-FDG PET/CT and HRCT from October 2015 to January 2021. Thirteen patients with solitary pulmonary intravascular metastasis were enrolled, including 8 women and 5 men aged 30–74 y (mean age, 54.1 ± 10.8 y). The demographic and clinical data of patients are listed in Table 1. This study was approved by the review boards for clinical investigation. All of the methods were performed in accordance with the Declaration of Helsinki and the relevant guidelines. Due to the retrospective nature of the study, informed consent was waived.

### 2.2. Reference Standard

Inclusion and diagnostic criteria of solitary pulmonary intravascular metastasis are as follows:

(1) Histopathological diagnosis
(2) Imaging and follow-up diagnosis are as follows:
  - Lesion(s) in the lung with ^18^F-FDG uptake higher than the background on PET/CT images
  - Lesion(s) located in the pulmonary vessel traveling area confirmed by HRCT and three-dimensional reconstruction images of the lung
  - Besides the primary lesions, the lung was the only involved organ
  - During the follow-up, lesion(s) showed tumor-like progressive enlargement with/without metabolic progressive increase
(3) Patients with the following situations were excluded:
  - Pulmonary tumor thrombosis caused by direct invasion of pulmonary or mediastinal tumors
  - Suffering from other diseases that can cause pulmonary vessel hypermetabolism (such as vasculitis)
  - Lesions were difficult to determine whether they were located in the pulmonary vessel traveling area or not
  - Distant metastases of organs other than the lung
  - Incomplete clinical or follow-up data

### 2.3. PET/CT and HRCT Scan

Patients were fasted for at least 6 h with the blood glucose level below 8 mmol/L, ^18^F-FDG (0.1 mCi/kg) was injected intravenously, and PET/CT (Ingenuity TF, Philips, Netherlands and Big Bore TF, Philips, Netherlands) scan was performed after 50–60 min of supine rest. CT scan for attenuation correction was performed first, using an automatic whole-body low-dose scan (an X‐ray tube current of 43 mAs, a tube voltage of 100 kV, and a spiral pitch factor of 1) from cranial apex to midfemur. PET scan was obtained at 9 to 10 couch positions, with an acquisition time of 1.0 min per position. Lung HRCT scans were performed with breath-hold after the PET/CT scan. The acquisition was performed at 120 kVp with 300 mAs. Images were reconstructed as contiguous 1 mm slices. Lung HRCT was conducted immediately after the PET scan without patient repositioning, and the bone algorithm was used. Analysis was performed on at least two reconstruction planes (usually axial and coronal reconstruction).

Follow-up chest CT examinations were performed using a variety of helical scanners, as different hospitals and different CT scanners were involved in this study. All examinations were performed with 16 to 128 detector row CT systems. CT acquisition parameters were 0.625–2.5 mm section thickness, 0.9–1.75 pitch, 120 kV, 80–350 mAs, or automatic tube current adjustment. Image reconstruction included contiguous 1–2 mm thick sections with high-resolution and standard algorithms for evaluation of the lung parenchyma and mediastinum. Patients were examined using the single breath-hold technique.

### 2.4. Image Analysis

Three experienced doctors—Reader 1: attending with 14 years of PET-CT diagnosis, reader 2: attending with 9 years of PET-CT diagnosis, and reader 3: attending with 20 years of chest imaging—analyzed the images of these patients and reached a consensus. The hypermetabolic lesions on ^18^F-FDG PET/CT were tracked continuously on the thin-layer and three-dimensional reconstruction images of the chest HRCT to identify the lesions located in the vascular running area, accompanied by the morphological changes of the corresponding blood vessels. If two or more diagnosticians could not confirm that the hypermetabolic lesions were located in the vascular area, the case was excluded in this study.

We conducted a visual and quantitative analysis of solitary pulmonary intravascular metastasis. The pattern of FDG uptake was evaluated by visual analysis, and the morphology was described using lung HRCT and three-dimensional reconstruction. For quantitative analysis, the SUVmax and length of hypermetabolic metastasis were measured.

### 2.5. Statistical Analysis

All the statistical analyses were performed using IBM SPSS Statistics version 24.0. The normal distribution of continuous variables was evaluated by the Shapiro–Wilk test. Data with normal distribution were expressed as mean ± standard deviation. Data with nonnormal distribution were expressed by the median.

## 3. Results

A total of 13 patients with 17 pulmonary intravascular metastases were found in the 18143 enrolled cancer patients. The incidence rate was 0.07%. Nine patients had one pulmonary intravascular metastasis, and 4 patients had two intravascular metastases, of which 2 patients' metastatic focus located in bilateral lungs. The primary tumors were lung cancer (3 cases), cervical cancer (3 cases), esophageal cancer (2 cases), pancreatic cancer (2 cases), breast cancer (1 case), gastric cancer (1 case), and rectal cancer (1 case). All primary tumors were confirmed by pathology. Among the 17 pulmonary intravascular metastases, 4 were confirmed by pathology, and the other 13 cases were diagnosed by imaging follow-up. Two patients appeared to have respiratory symptoms: cough and asthma, and the other 11 patients had no obvious respiratory symptoms (Table 1).

**Figure 1:**
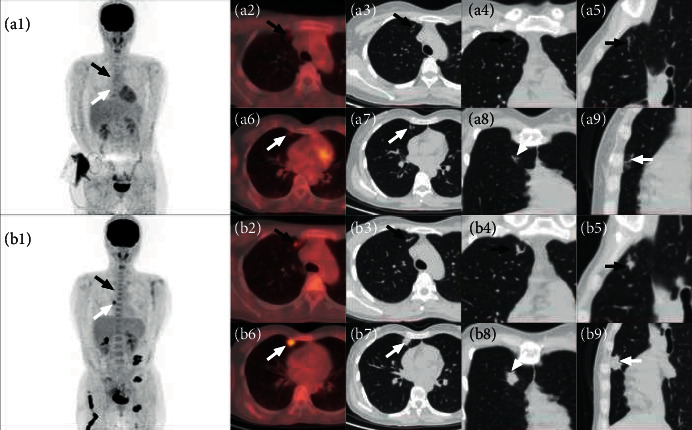
A 30-year-old woman with cervical cancer underwent surgery. ^18^F-FDG PET/CT (a1-2 and a6) scan showed an increased uptake of FDG in the right upper lobe of the lung subpleura. The SUVmax were 1.5 (white arrow) and 1.2 (black arrow), respectively. HRCT (a3-5 and a7-9) revealed corresponding branch-like enlargement of pulmonary blood vessels. After 6 months of follow-up of ^18^F-FDG PET/CT (b1-2 and b6) and HRCT (b3-5 and b7-9), two lesions were found to be enlarged in volume and increased in metabolism. The SUVmax were 6.5 and 3.0, respectively. The lesions below (shown by white arrow) were enlarged as masses.

**Figure 2:**
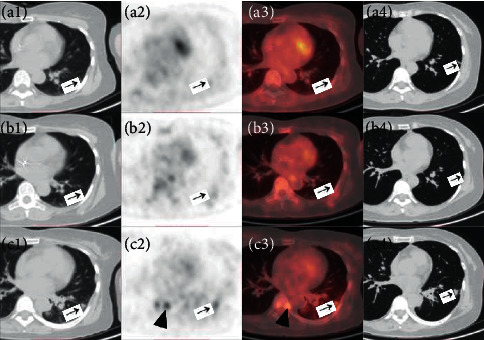
A 45-year-old female with rectal cancer underwent surgery. ^18^F-FDG PET/CT (a1-3) scan showed an increased FDG uptake in the left lower lobe of the lung (black arrow). (SUVmax = 1.9). HRCT (a4) revealed a strip-like thickening of the corresponding pulmonary vessel. The patients received systemic antineoplastic therapy and were reexamined 3 months later. PET/CT (b1-3) and HRCT (b4) scans showed that the pathological pulmonary vessel and metabolism basically returned to normal. After 11 months, ^18^F-FDG PET/CT (c1-3) and HRCT (c4) found that the pulmonary vessel was markedly enlarged with increased metabolism, and new bone metastasis appeared (black triangle) (SUVmax = 3.5).

The SUVmax of 17 solitary pulmonary intravascular metastases ranged from 1.1 to 5.4, with an average of 2.4 ± 1.4, and the length of hypermetabolic metastases ranged from 5.1 mm to 24.1 mm, with an average of 10.7 ± 6.5 mm. The morphology features of solitary pulmonary intravascular metastasis were analyzed by lung HRCT continuous tomography and three-dimensional reconstruction. All 17 solitary pulmonary intravascular metastases were located in the distal pulmonary vessels, and none was involved in the pulmonary vessel above the segment (Figures 1 and 2). Fifteen solitary pulmonary intravascular metastases showed strip or branched pulmonary vessels enlargement with increased metabolism, and the other 2 cases only showed strip metabolic enhancement without pulmonary vascular abnormalities. The 13 solitary pulmonary intravascular metastases diagnosed by follow-up showed progressive thickening of involved vessels, of which 7 showed increased metabolism on FDG PET/CT (the other 6 were only followed up by chest CT) (Figures 1 and 2). Besides, 4 patients had a recurrence of tumors in the original surgical site, 7 patients had regional lymph node metastases, and 4 patients had new metastases of organs.

## 4. Discussions

Solitary pulmonary intravascular metastasis is a rare form of lung metastasis. Shepard et al. preliminarily summarized the imaging manifestations of pulmonary intravascular metastasis for the first time after the validation of autopsy [10]. However, their study only involved the CT manifestations of advanced pulmonary intravascular metastasis. Owing to the aggressive and poor prognostic nature of lung metastasis, early identification of pulmonary intravascular metastasis has a higher clinical value. All the 13 patients included in this study were solitary pulmonary intravascular metastasis without metastasis of other organs. In order to ensure the accuracy of diagnosis, patients in this study had multiple follow-up images, and these follow-ups were based on the needs of diagnosis and treatment of tumor patients, without additional costs and radiation.

In this study, the incidence of solitary pulmonary intravascular metastasis was 0.07%, but the incidence would be significantly underestimated. Mainly for the following reasons: (1) the patients included in the study required follow-up images, and many patients were excluded because of incomplete data; (2) many pulmonary intravascular metastases decrease or disappear under antitumor therapy and cannot be distinguished from other benign pulmonary lesions; and (3) solitary pulmonary intravascular metastasis is an early disease, which can be missed easily because of inconspicuous performance on FDG PET/CT. An accurate incidence rate requires a more rigorous trial design. In the study, only 2 patients appeared to have respiratory symptoms: cough and asthma, who had a long medical history, so solitary pulmonary intravascular metastasis is hard to be diagnosed by clinical manifestations alone, which was always ignored by clinicians.

The 13 patients included in the study were incidentally found during follow-up and did not receive additional antineoplastic therapy. During the follow-up, in addition to the progress of pulmonary intravascular metastases, 4 had a recurrence of tumors in the original surgical site, 7 had regional lymph node metastases, and 4 had new metastases of organs. Therefore, the authors speculated that solitary pulmonary intravascular metastasis may be an indicator to predict tumor progression, and early clinical intervention may improve the prognosis of patients, which requires more cases and longer follow-up for validation.

In this study, 9 patients had one pulmonary intravascular metastasis, and 4 patients had 2 intravascular metastases, of which 2 patients' metastatic focus was located in bilateral lungs. It means solitary pulmonary intravascular metastasis can be multiple and also can affect both lungs. Our study showed that all the pulmonary intravascular metastases were located in the terminal pulmonary vessels and no metastasis was found in the segmental or lobar pulmonary vessels, which may be associated with the high blood flow rate making the tumor cell difficult to stay. Other studies have similar results [10–12]. Compared with proximal pulmonary vessels, distal pulmonary vascular abnormalities were more easily missed by traditional imaging (even enhanced contrast CT scan of the pulmonary vessel). Therefore, it is necessary to carefully observe the metabolic changes in the peripheral lung tissue during FDG PET/CT scanning in tumor patients in order to avoid missed diagnosis. We found that 15 metastases showed strip or branched pulmonary vessels enlargement with increased metabolism and the other 2 cases only showed strip metabolic enhancement without pulmonary vascular abnormalities. We speculated that it may be related to the development of pulmonary intravascular metastasis. In the early stage, the tumor cells grew along the vascular endothelium, the vascular morphology was generally normal, and only the metabolism was increased. With the proliferation of tumor cells, the blood vessels thickened unevenly and it could be bifurcated when many adjacent vessels were involved, which can then be detected by CT. This was consistent with the result found by Erhamamci et al. [7]. The growth and proliferation of tumor cells will result in increased ^18^F-FDG uptake at the corresponding site, and PET can detect this change more sensitively than CT before the morphological changes of blood vessels [12].

In this study, the range of SUVmax was 1.1 to 5.4, with an average of 2.4 ± 1.4. Xi et al. systematically summarized previous studies on benign and malignant pulmonary vascular lesions. They found that there were significant differences between them, and malignant intravascular lesions had a higher metabolic level [9]. The abnormal hypermetabolism of tumor metastasis may be related to the tumor cell itself and dual blood supply of tumor metastasis (tumor vessels and pulmonary arteries). Some intravascular benign lesions (such as thromboembolism) can also cause hypermetabolism, but the affinity of FDG was lower than that of intravascular malignant lesions [6, 13, 14]. Xi et al. also found that sensitivity, specificity, and accuracy of ^18^F-FDG PET/CT in differentiating benign and malignant pulmonary vascular lesions were 98.4%, 96.8%, and 97.8%, respectively, with SUVmax = 3.3 as the cutoff. However, the SUVmax of solitary pulmonary intravascular metastasis in this study was low, and most of them were lower than this cutoff. By comparing the results of Xi et al., we found that the reasons for this difference may be as follows: (1) we excluded the direct invasion of the pulmonary vessel made by pulmonary or mediastinal tumors, thus avoiding the influence of primary tumors on tumor thrombus, but the metabolism of primary tumors was significantly higher than that of pulmonary intravascular metastasis; and (2) pulmonary intravascular metastasis in other studies was mostly located above the segmental vessel, while the involved arteries were all distal pulmonary arteries in this study, which resulted in smaller volume and lower metabolism. Although the metabolism of solitary pulmonary intravascular metastasis in this study was low, it can be well differentiated from other intravascular lesions by metabolism, morphology, and multiple follow-ups.

In the diagnosis of solitary pulmonary intravascular metastasis, the combination of ^18^F-FDG PET/CT and lung HRCT is very important. Because solitary pulmonary intravascular metastasis occurs in the small-vessel lumen and travels in accordance with the normal pulmonary vessel, it is often confused with normal pulmonary texture on attenuation-corrected CT images, which is easily missed by radiologists. This was also confirmed by this group of cases. At the same time, the respiratory movement often cause the mismatch of PET and attenuation-corrected CT images, so it is necessary to hold breath during lung HRCT, and three-dimensional reconstructed images are used to locate the lesions, to further determine whether the lesions were located in the pulmonary vessel and to differentiate them from other morphologically similar lesions (such as fibrous stripes and bronchial lesions). Some inflammatory diseases may appear with high metabolism of FDG, but they do not locate in the vascular area, and there is no thickening of corresponding vessels (such as tracheitis and inflammatory cords). However, a few of the distal inflammatory cords and vasculitis may be closely related to the adjacent blood vessels and also accompanied by increased metabolism. At this time, it may be difficult to distinguish by relying solely on FDG PET/CT and HRCT, and the diagnosis must depend on follow-up. The morphological and metabolic changes can be observed in the follow-up images: the inflammatory diseases do not appear as the corresponding vascular thickening and metabolic increase of the intravascular metastasis progresses. The main purpose of this study was to describe the imaging performance of solitary pulmonary intravascular metastasis, not to conduct epidemiological studies. In order to ensure the accuracy of the diagnosis, some controversial cases were excluded from this study. Unlike other studies, contrast-enhanced CT (CECT) scans were not performed in the present study. On the one hand, one could not observe the filling of the terminal pulmonary vessel very well on CECT; on the other hand, other studies suggested that CECT was unnecessary because of its limited value in distinguishing between benign and malignant intravascular lesions [15].

Here are some limitations in our study: firstly, limited by the FDG PET/CT resolution, minimal lesions with a diameter of less than 4 mm may not be detected. Secondly, some tumors—such as signet ring cell carcinoma of the gastrointestinal tract and mucinous adenocarcinoma—have a low uptake of ^18^F-FDG, which could affect the detection of pulmonary intravascular metastasis. Thirdly, the diagnosis of most solitary pulmonary intravascular metastasis in this study was based on clinical and imaging follow-up. Although the duration of the follow-up was sufﬁciently long, and all patients demonstrated a signiﬁcant change to allow for a definite diagnosis, misdiagnosis was inevitable. Finally, the rarity of solitary pulmonary intravascular metastasis and the small number of cases made selection bias inevitable.

In conclusion, solitary pulmonary intravascular metastasis is a rare complication of malignant tumors that lacks specific clinical symptoms. Combined ^18^F-FDG PET/CT with lung HRCT—FDG hypermetabolism in the distal pulmonary vessel with stripe-like or branched changes of the corresponding pulmonary vessel—could indicate the solitary pulmonary intravascular metastasis. If in doubt, it can be confirmed by follow-up or pathology.

## Figures and Tables

**Table 1:** Demographic, clinical data, and imaging data of 13 patients with pulmonary tumor thrombosis.

Patient no.	Age (y)	Sex	Primary tumor	Respiratory symptoms	Metastasis no.	Location	SUVmax	Length (mm)	Pulmonary vascular morphology	Follow-up
1	58	F	Lung	None	1	RUL	1.1	5.2	Normal	Lymphatic metastasis
2	57	M	Lung	Cough	1	LLL	3.2	11.8	Thickening	Tumor recurrence and organ metastasis
3	53	M	Lung	None	1	RUL	2.7	24.1	Thickening	No tumor recurrence or new metastasis
4	57	F	Cervical	None	2	RUL/LUL	5.4/4.3	22.7/23.7	Thickening	Tumor recurrence
5	45	F	Cervical	None	1	RUL	1.2	6.2	Thickening	Lymphatic metastasis
6	30	F	Cervical	None	2	RUL/RUL	1.5/1.2	5.1/7.4	Thickening	No tumor recurrence or new metastasis
7	45	F	Rectal	None	1	LLL	1.9	9.8	Thickening	Lymphatic metastasis
8	38	F	Breast	None	1	RML	1.3	8.2	Thickening	Organ metastasis
9	74	M	Esophageal	Asthma	2	RUL/LLL	3.0/2.0	9.9/7.1	Thickening	Lymphatic metastasis and organ metastasis
10	59	M	Esophageal	None	1	LUL	1.1	6.2	Normal	Tumor recurrence and lymphatic metastasis
11	60	F	Gastric	None	1	LUL	3.8	13.2	Thickening	Lymphatic metastasis
12	59	M	Pancreatic	None	2	RUL/RUL	1.1/1.3	5.2/6.6	Thickening	Organ metastasis
13	59	F	Pancreatic	None	1	RLL	4.1	9.1	Thickening	Tumor recurrence and lymphatic metastasis

F, female; M, male; RUL, right upper lobe; RML, right middle lobe; RLL, right lower lobe; LUL, left upper lobe; LLL, left lower lobe.

## Data Availability

The datasets generated and/or analyzed in the current study are available from the corresponding author on reasonable request.
